# Comparative clinical trials in psychotherapy: Have large effects been replicated?

**DOI:** 10.1017/S2045796020000402

**Published:** 2020-05-15

**Authors:** Nickolas D. Frost, Thomas W. Baskin, Bruce E. Wampold

**Affiliations:** 1 Department of Counseling Psychology, University of Wisconsin-Madison, Madison, WI, USA; 2 University of California, College of Biological Sciences, Davis, CA, USA; 3 Modum Bad Psychiatric Center, Vikersund, Norway

**Keywords:** Depression, psychotherapy, randomised controlled trials, research design and methods

## Abstract

**Aims:**

The purpose of this review is to examine the replication attempts of psychotherapy clinical trials for depression and anxiety. We focus specifically on replications of trials that exhibit large differences between psychotherapies. The replicability of these trials is especially important for meta-analysis, where the inclusion of false-positive trials can lead to erroneous conclusions about treatment efficacy.

**Methods:**

Standard replication criteria were developed to distinguish direct from conceptual replication methodologies. Next, an exhaustive literature search was conducted for published meta-analyses of psychotherapy comparisons. Trials that exhibited large effects (*d* > 0.8) were culled from these meta-analyses. For each trial, a cited replication was conducted to determine if the trial had been subsequently replicated by either ‘direct’ or ‘conceptual’ methods. Finally, a broader search was conducted to examine the extent of replication efforts in the psychotherapy literature overall.

**Results:**

In the meta-analytic search, a total of *N* = 10 meta-analyses met the inclusion criteria. From these meta-analyses, *N* = 12 distinct trials exhibited large effect sizes. The meta-analyses containing more than two large effect trials reported evidence for treatment superiority. A cited replication search yielded no direct replication attempts (*N* = 0) for the trials with large effects, and *N* = 4 conceptual replication attempts of average or above average quality. However, of these four attempts, only two partially corroborated the results from their original trial.

**Conclusion:**

Meta-analytic reviews are influenced by trials with large effects, and it is not uncommon for these reviews to contain several such trials. Since we find no evidence that trials with such large effects are directly replicable, treatment superiority conclusions from these reviews are highly questionable. To enhance the quality of clinical science, the development of authoritative replication criteria for clinical trials is needed. Moreover, quality benchmarks should be considered before trials are included in a meta-analysis, or replications are attempted.

## Introduction

There are few concepts more vital to the integrity of a scientific discipline than replication. Put simply, replication involves re-testing a hypothesis to corroborate a scientific result (Schmidt, 2009). Replication functions as the final arbiter of scientific knowledge – forcing scientists to refine (or discard) flawed theories that cannot precisely predict the outcome of successive experiments (Francis, 2012).

Replication is especially important in clinical sciences, where failure to reproduce scientific results can lead to the dissemination of ineffective clinical practices (Prasad *et al*., 2013). In both medicine and psychiatry, reproducibility in clinical science has been investigated (Ioannidis, 2005; Tajika *et al*., 2015) but unfortunately, not in psychotherapy research (Tackett *et al*., 2019).

Based on thousands of clinical trials and hundreds of reviews of those trials, it is incontrovertible that psychotherapy is an effective intervention across a wide range of mental health problems (Lambert, 2013; Wampold and Imel, 2015; Munder *et al*., 2019; Cuijpers *et al*., 2019*a*), and replications to address the question of absolute efficacy would merely provide redundant information. However, there are ongoing questions about the superiority of particular psychotherapies (Wampold, 2005; Tolin, 2014; Wampold *et al*., 2017; Cuijpers *et al*., 2019*b*).

There is little scientific consensus about what constitutes a meaningful superiority result in psychotherapy research. Occasionally, however, a single psychotherapy comparison or a few of these comparisons produce *large* effects (Cohen, 1988). These trials can influence meta-analytic conclusions regarding treatment superiority, and in turn, treatment guidelines (Cottraux *et al*., 2000; Clark *et al*., 2006; Wampold *et al*., 2017). The replicability of these trials is important because large effects might well be false-positive results.

The purpose of the present study was to examine the replications of psychotherapy trials demonstrating large treatment differences. We focused on trials for depression and anxiety-related disorders because they are among the most prevalent (Kessler *et al*., 2005*a*, 2005*b*), and many psychotherapies for these disorders are classified as having *strong* empirical support (see https://www.div12.org/psychological-treatments/frequently-asked-questions/).

### Ingredients of replication

Schmidt (2009) distinguished two broad categories of scientific replication: *direct* and *conceptual.* Generally, a direct replication involves procedural duplication of an earlier experiment, whereas a conceptual replication involves testing the central hypothesis of the earlier study through alternative experimental arrangements (Schmidt, 2009). As the ‘gold standard’ of experimental designs, randomised clinical trials (RCTs) are well suited for direct or conceptual replication, but a direct replication offers the strongest corroboratory evidence because it most closely resembles the original trial (Schmidt, 2009). Alternatively, conceptual replication may well elucidate other aspects of the phenomenon, such as generalizability.

The experimental logic of a clinical trial is standard across scientific disciplines. In the case of psychotherapy comparisons, a sample of patients suffering from a particular disorder are recruited, randomly assigned to two or more treatment conditions, receive the respective treatments, and are evaluated at termination to compare outcomes between treatment groups (Heppner *et al*., 2008). Better outcomes for the patients in one treatment condition *vis-à-vis* the outcomes for the other treatment, beyond what is expected by chance, are evidence for the superiority of that treatment (Heppner *et al*., 2008).

Despite this sound logic, inferences regarding treatment superiority (i.e. Treatment A is superior to B) in psychotherapy trials are more complicated. Blinding is not possible in psychotherapy, and researcher allegiance to a particular treatment can influence outcomes in direct comparisons (McLeod, 2009; Munder *et al*., 2011, 2013). Moreover, unlike medicine, psychotherapies cannot be completely standardised across patients or administrations. In psychotherapy, the therapist delivering the treatment, regardless of the degree to which the treatment is standardised, can make a difference; that is, therapists are not interchangeable (Baldwin and Imel, 2013). Consequently, therapist characteristics (training, experience, etc.) and representativeness should be considerations for replication.

Despite these complexities, enough methodological features can be controlled to discriminate direct and conceptual replication of a psychotherapy trial. In a direct replication, nearly all trial features must be the same between the original trial and replication attempt (denoted by ‘ + ’ in Table 1). These features broadly include duplication of treatment delivery, therapist expertise, characteristics of the patient sample and outcome assessment.

**Table 1.** Direct replication criteria

		Direct	Conceptual
Treatments			
General Tx	Same treatment	+	+
Dose	Same number of sessions	+	−
Format	Same format (Group *v*. Individual)	+	−
Manual	Same Tx manuals	+	+
Clients			
Diagnosis	Same diagnostic population	+	+
Characteristics	Similar demographic population	+	−
Sample size	At least as large	+	+
Therapists			
Competence	Same profession/training	+	−
Adherence	Protocol checks – same level	+	−
Outcome	Same outcome measures	+	−
Design			
Randomization	Random assignment	*+*	+
Conclusion			
Effect size	Same or larger Tx difference	+	+

‘+’ = This is a feature that must remain the same in the primary trial and replication; ‘−’ = this is a feature that can be different in the replication.

Some trial features, however, can be altered so that clinical trial could qualify as a conceptual replication when the researcher is interested in examining parametric effects or generalizability across contexts. The number of psychotherapy sessions delivered (treatment dose), format of treatment (i.e. individual or group; see Table 1) and context (e.g. university speciality clinic *v*. community clinic) are examples of features that may vary between the primary trial and replication attempt in these situations. A characteristic of a conceptual replication is that specific features of the original study are intentionally varied to examine a particular conjecture. A case could be made, however, that to accept any superiority result as an established scientific finding, at least one direct replication is required (Schmidt, 2009).

## Method

This replication investigation employed the following steps. First, meta-analyses of comparative psychotherapy treatments for depression and anxiety were identified. Second, from those meta-analyses, trials with large effects (*d* > 0.8) were culled from the meta-analyses. Third, all citations to the identified trials were examined to determine whether they qualified as direct or conceptual replications according to the components listed in Table 1. These processes are different from canonical reviews and therefore various standards (e.g. PRISMA) were not applicable. Although these procedures were determined *a priori*, the research was not registered.

### Identification of meta-analyses

Meta-analyses were only included in this study if they met the following criteria: (a) they included RCTs that directly compared at least two active psychosocial treatments, (b) patients were adults who suffered from a diagnosable anxiety or depressive psychiatric condition as defined by any edition of the Diagnostic and Statistical Manual of Mental Disorders or other accepted diagnostic nosologies, and (c) they were published in a peer-reviewed journal.

The search for meta-analytic reviews included an exhaustive search of the database PsycINFO using the search engine ProQuest by two of the authors (BLIND). Primary search terms included ‘Depression’, ‘Anxiety’ and ‘Meta-analysis’. Filtering options for adult population and peer-reviewed journal were applied to limit search results. Potential meta-analyses were evaluated by all the authors to determine whether they met the eligibility criteria until full consensus was reached.

### Large effect trials

A clinical trial database was developed that included all the comparative trials contained in the meta-analyses. Some meta-analyses included only comparative trials; for other meta-analyses, comparative trials formed a subset of trials. In the latter situation, only the comparative trials were included in this study. Some trials were contained in more than one meta-analysis and these duplicate trials were noted and then excluded so that a trial only appeared once in the trial database.

Effect size conventions (Cohen, 1988) stipulate that small effect sizes include those in the range of 0.2, medium effect sizes are in the range of 0.5 and large effects are in the range of 0.8. To identify the largest effect trials from our database, we used a cut-off value of *d* = 0.80. Trials that exhibited absolute effect sizes equal to or greater than this cut-off were included in a new dataset that only contained large effect trials. Only targeted outcomes that measured diagnosis-specific symptomology were considered in this investigation, as reported in the published meta-analysis or supplied from the meta-analytic authors upon our request in two cases (Tolin, 2010; Cuijpers *et al*., 2016).

Finally, trials were excluded that were not ‘bona-fide’ psychotherapy comparisons (Wampold *et al*., 1997). The bona-fide criteria were interpreted liberally; we only eliminated comparisons to treatment-as-usual conditions and comparisons to ‘non-active’ control conditions, as identified by the trials' authors. The large effect trials were graded on their overall methodological quality by two independent raters (two of the authors) using the Psychotherapy Quality Rating Scale (PQRS) (Kocsis *et al*., 2010). This scale was developed for RCTs of psychotherapy and has demonstrated good internal consistency in validation studies (*α* = 0.87). The scale includes 24 items that range from 0 to 2, examining six trial quality domains: definition and delivery of treatment, patient description, outcome assessment, appropriateness of data analysis, treatment assignment and overall quality. The final item ranges from 1 to 7, where a score of 1 indicates an *exceptionally poor* trial and 7 indicates an *exceptionally good* trial. Thus, the PQRS ranges from 1 to 55, with higher scores indicating a higher quality trial.

### Replication searches

The first step in locating replications was based on the premise that a replication would cite the trial it was replicating. Accordingly, we used the Web of Science to search all citations of each large effect trial in our database. The number of citations for each trial is shown in Table 3. Presumably, a replication would use the word *replication* in the title or abstract, which we coded. However, partly because only one citation to any of the trials used the term replication in this way, we did not limit the identification of replications to this standard. Rather, each study was coded by two independent raters (two authors) where we applied the criteria in Table 1 to determine if each citation was a replication of a previous trial. If either rater indicated that a trial was a possible replication, we included that trial in our results.

After completing this process, we were concerned that we may have missed replication attempts of trials in our database. Thus, in a *post-hoc* search, we used two key words (*Psychotherapy*, *Replication*) to search the Web of Science and PsycINFO databases for any replication attempt of a psychotherapy clinical trial as identified by its title. Results were filtered only by ‘clinical trial methodology’ from the list of methodologies. An advantage of this secondary search was that it allowed us to assess the extent of replication efforts in the psychotherapy literature writ large.

## Results

### Meta-analyses

**Fig. 1.**
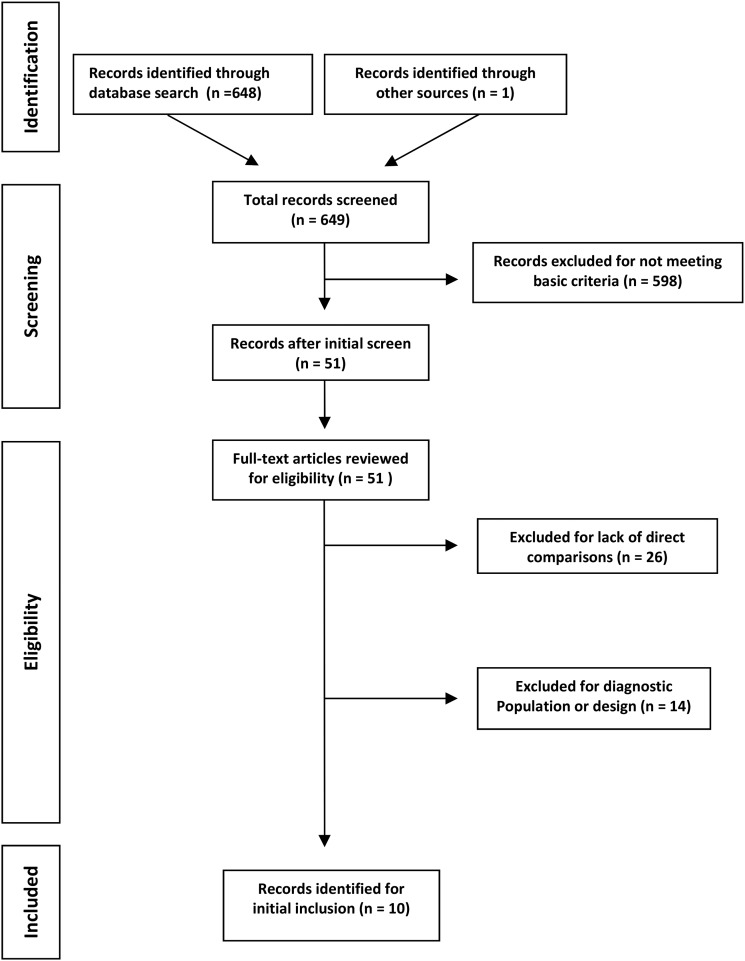
Flow diagram of the meta-analysis selection process.

Search results for the meta-analyses returned 648 ‘hits’ from PsycINFO. In total, 638 meta-analyses were excluded for the lack of direct comparisons, the inclusion of non-RCT research designs or the lack of patient samples with a defined disorder (see Fig. 1). Ultimately, ten meta-analyses met all inclusion criteria and composed the meta-analysis database (Table 2).

**Table 2.** Meta-analyses of direct treatment comparisons

Meta-analysis	ID	Disorder	Direct comparisons	Aggregate effect (95% CI)	*N* influential trials	Conclusion
Baardseth *et al*. (2013)	1	Depression and Anxiety	13	0.14 [−10.08 to 0.35]	2	*These analyses, in combination with previous meta-analytic findings, fail to provide corroborative evidence for the conjecture that CBT is superior to bona fide non-CBT treatments. (pp. 395)*
Cuijpers *et al.* (2006)	2	Late-life depression	12	Not reported	2	*No clear differences in effects between different psychological treatments were found (pp. 1145).*
Cuijpers *et al.* (2010*b*)	3	Chronic major depression	4	0.15 [−0.25 to 0.55]	1	*The mean effect size indicating the difference between IPT and other psychotherapies was small (pp. 58)*
Cuijpers *et al*. (2012)	4	Depression	30	−0.20 [−0.32 to 0.08]	2	*NDST was less effective than other psychological treatments (pp. 280)*
Cuijpers *et al.* (2016)	5	Depression	7	0.29 [0.01–0.56]	4	*CBT was found to be more effective than other therapies in older adults (0.29), University students (0.51) in patients with comorbid addictive disorders, and in university students. (pp. 975)*
Keefe *et al*. (2014)	6	Anxiety disorders	13	0.02 [−0.21 to 0.26]	2	*PDT did not differ significantly from alternative treatments (pp. 309)*
Lilliengren *et al*. (2016)	7	Depression and anxiety	14	0.01 [−0.13–0.15]	0	*We found no differences between EDT and active treatments (e.g. medication, CBT, manualized supportive therapy) at posttreatment, but EDT outperformed supportive therapy at follow-u (pp. 90)*
Newby *et al*. (2015)	8	Anxiety and depressive disorders	4	0.58 [0.03–1.16]	1	*Preliminary evidence from 4 comparisons with disorder-specific treatments suggests that transdiagnostic treatments are as effective for reducing anxiety and may be superior for reducing depression. (pp. 91)*
Tolin (2010)	9	Depressive and anxiety disorders	32	0.22 [0.09–0.35]	4	*These results argue against previous claims of treatment equivalence and suggest that CBT should be considered a first-line psychosocial treatment of choice, at least for patients with anxiety and depressive disorders. (pp. 710)*
Tolin (2014, 2015)	10	Depression and anxiety	13	0.23 [−0.01 to 0.6]	2	*Patients receiving and completing CBT fare significantly better at posttreatment than do patients receiving and completing other psychotherapies (pp. 357)*

CBT, cognitive behavioural therapy; PDT, psychodynamic therapy; NDST, non-directive supportive therapy; IPT, interpersonal therapy; EDT, experiential dynamic therapy.

*Note:* all effect sizes are reported for targeted symptom measures.

Of the ten meta-analyses, four examined populations suffering from depression, five focused on both depression and anxiety-related disorders and one examined anxiety. Aggregate effect size estimates ranged from 0.02 to 0.46 in these meta-analyses, and five of the ten reported finding evidence for treatment superiority (see Table 2). An important caveat relates to Tolin's (2014) meta-analysis which found cognitive-behaviour therapy (CBT) superior to other psychotherapies for anxiety disorders as this conclusion was corrected in a subsequent corrigendum due to a calculation error contained in the earlier analysis (n.b., we used the corrected effect sizes in this study) (Tolin, 2015).

### Clinical trials

Clinical trials drawn from these meta-analyses spanned the years 1972–2016, totalling 137 unique trials with 157 treatment comparisons – involving CBT, dynamic therapies, behavioural activation, cognitive therapy (CT), interpersonal therapy and emotion-focused therapies. Effect sizes from all trials ranged from *d* = 0.0 to *d* = 1.56 on targeted outcomes, with a mean average effect of *d* = 0.41 (s.d. = 0.32). Approximately 57% (*k* = 88) of the comparisons yielded small-to-medium effects, 34% (*k* = 56) yielded medium-to-large effects, and 9% (*k* = 14) of the comparisons exhibited large effects greater than or equal to the *d* = 0.8 cut-off used in this study. These large effect sizes came from 12 distinct clinical trials (see Table 3). It is important to note that five of the large effect trails appeared in more than one of our meta-analyses.

**Table 3.** Clinical trials with the largest effect sizes

Trial	Meta ID	Diagnosis |ES|	Treatments *A* > *B*	Quality	PQRS total	Citations #	Direct attempts	Conceptual attempts
Ayen and Hautzinger (2004)	4, 5	Depression 1.56	CBT > SUP	*	*	9	0	0
de Jong *et al*. (1986)	3	Depression, Dysthymia 1.03	CBT > SUP	*Moderately poor*	24	1	0	0
Durham *et al*. (1994)	1, 6, 9, 10	GAD 0.9	CT > DYN	*Average*	31	101	0	3
Gallagher-Thompson and Steffen (1994)	9, 5	Depression 1.27	CBT > DYN	*Moderately good*	37	126	0	3
Klausner *et al*. (1998)	2	Depression 1.4	GFT > REM	*Very poor*	18	57	0	1
Latour and Cappeliez (1994)	2	Depression 0.92	CBT > CT	*Moderately poor*	22	16	0	1
Milrod *et al*. (2007)	6	Anxiety 0.89	DYN > AR	*Very good*	43	147	0	0
Norton (2012)	8	Anxiety, Depression 1.54	TRN > RLX	*Moderately good*	37	72	0	0
Shapiro *et al*. (1994)	9	Depression 1.44	CBT > DYN	*Very good*	41	268	0	2
Shaw (1977)	4, 5	Depression		*Very poor*	18	200	0	5
		1.13	CT > BAT					
		0.94	BAT > SUP					
Shear *et al*. (2001)^a^	1, 9, 10	PD 0.8	CBT > EFT	*Moderately poor*	26	38	0	0
Taylor and Marshall (1977)	5	Depression 0.89	CBT > BAT	*Exceptionally poor*	16	0	0	0

CBT, cognitive behaviour therapy; CT, cognitive therapy; DYN, dynamic therapy; REM, reminiscence psychotherapy; EFT, emotion-focused therapy; TRN, transdiagnostic therapy; SUP, supportive therapy; BAT, behavioural activation therapy; RLX, relaxation therapy; GFT, goal-focused therapy; CT, cognitive therapy; AR, applied relaxation, quality ratings were averaged between two independent raters; GAD, generalised anxiety disorder; PD, panic disorder.

a Manual not available.

*Trial unavailable in English. Quality = item 25 from the PQRS.

#### Large effect trials

Treatment comparisons from the large effect trials included CBT, interpersonal therapy (IPT), psychodynamic psychotherapies (DYN), transdiagnostic therapies (TRN), manualised supportive psychotherapies (SUP) and behavioural activation (BAT). The average number of patients per treatment condition was 21.8 and the average number of psychotherapy sessions delivered for each treatment condition was 13.6. There were no systematic differences between superior and inferior treatments in terms of sample size or treatment dose in these trials. Effect sizes at post-treatment on targeted outcomes (e.g. Beck Depression Inventory (BDI) for depression) ranged from *d* = 0.8 to *d* = 1.56, with a mean effect of *d* = 1.1 (s.d. = 0.25).

The index of rater agreement for trial quality ratings was good (ICC = 0.83) between the two raters (Borenstein *et al*., 2009), and the final PQRS scores were the average of the two (Table 3). The ratings on the PQRS ranged from 16 to 43 out of the possible score of 55. No large effect trials were rated as *exceptionally good* but two were rated as *very good*; over half were rated as *exceptionally poor*, *very poor* or *moderately poor*. Treatment comparisons in the lowest quality trials found CBT superior to behavioural activation (*d* = 0.89) for depressed adults (Taylor and Marshall, 1977); CT superior to behaviour therapy and supportive therapy (*d* = 1.13; *d* = 0.90, respectively) for depressed college students (Shaw, 1977); and goal-focused group psychotherapy superior to group reminiscence therapy (*d* = 1.14) for late-life depressed adults (Klausner *et al*., 1998). The highest quality trials found dynamic therapy superior to applied relaxation (*d* = 0.89) for adults with panic disorder (Milrod *et al*., 2007); and CBT superior to dynamic therapy (*d* = 1.44) (Shapiro *et al*., 1994). Trial characteristics that attenuated quality ratings included poor definition or execution of treatment delivery, and unclear description of patients and diagnostic classification.

### Replication search results

The large effect trials were cited 1035 times in the Web of Science (see Table 3), with an average number of 85 citations per trial. The least cited trial was (Taylor and Marshall, 1977) not cited at all in the Web of Science and the most (Shapiro *et al*., 1994) was cited 268 times. Of all the recorded citations, 126 (12%) were clinical trials.

Applying the criteria from Table 1 to each citing clinical trial, there were no trials that could be deemed a direct replication attempt in terms of their methodology. That is, no trials methodologically duplicated an original trial by comparing the same treatments (dose, format, manual), on the same population (diagnosis, patient characteristics), with therapists of the same training and experience, using an approximately similar measure of clinical outcome. In fact, only 15 of all citing trials screened included at least one general treatment from the original trial in an active comparison (Table 3). The absence of identical treatment comparisons made these trials only eligible as conceptual replication attempts (see online Supplementary Table S4).

From the 15 trials that could possibly be considered conceptual replications, treatment format and dose varied from the influential trial, which is acceptable in a conceptual replication whose purpose is generalizability. However, only six of these possible replications (Wilson *et al*., 1983; Barkham *et al*., 1996; Barkham *et al*., 1999; Mohr *et al*., 2001; Arntz, 2003; Gallagher-Thompson *et al*., 2003) used the same treatment manual as the original trial. The number of potential replication attempts was further reduced, because only four of these trials included an approximately similar diagnostic population of patients (Wilson *et al*., 1983; Barkham *et al*., 1996; Mohr *et al*., 2001; Arntz, 2003). From the remaining four trials, only one of them was explicitly identified as a replication attempt by its authors (Barkham *et al*., 1996). Characteristics of the replication attempts can be seen in online Supplementary Table S4.

Are any of these trials ‘successful’ replication attempts? Conclusions from two of the four quasi-valid conceptual replication attempts loosely corroborated the original trial. Mohr *et al*. (2001) compared 16 weeks of CBT to dynamic therapy and found a significant difference at post-treatment on the BDI in favour of CBT for depressed patients with multiple sclerosis. But this effect was notably smaller (*d* = 0.55 *v*. *d* = 1.27) than the post-treatment difference found in the original trial. Barkham *et al*. (1996) intentionally replicated Shapiro *et al*.'s (1994) trial and found general equivalence between CBT and dynamic therapy at post-treatment; however, significant differences favouring CBT observed on the BDI (*d* = 1.44)^1^ from the original trial were not replicated. The remaining two replication attempts reached divergent conclusions than the original trial they cited. For example, in direct contrast to Durham *et al*. (1994), Arntz (2003) found applied relaxation and CT to be equivalent after 12 sessions of treatment on composite measures of a generalized anxiety disorder (*d* = 0.14). Finally, Wilson *et al*. (1983) found small differences (*d* = 0.25) between CT and behavioural treatment following eight sessions of counselling, whereas Shaw (1977) found CT superior to behaviour therapy (*d* = 1.13). As a whole, these conceptual replications produced weaker or contradictory results compared to the original trial they cited.

Results from the general replication search resulted in 38 hits from our search terms *Replication* and *Psychotherapy.* Examining each hit revealed no direct or conceptual replication attempts of a psychotherapy comparison for adult depression or anxiety. More specifically, six studies were excluded because they examined a child population, ten did not include direct psychotherapy comparisons, and four were not clinical trials at all. The remaining 18 studies were either narrative reviews, comparisons to treatment-as-usual conditions or waitlists. One study was identified as a replication attempt, but not of a comparative psychotherapy result (Chambless *et al*., 2017).

## Discussion

We examined replication attempts of trials that exhibited large effects in published meta-analyses. The essential finding is that replications are scarce: There were no direct replication attempts, and only a few possible conceptual replication attempts, many of which failed to corroborate the conclusions of the original trial. Several large effect trials had poor quality ratings. However, even for the two trials rated as ‘very good’ quality (Shapiro *et al*., 1994; Milrod *et al*., 2007), there were no conceptual replication attempts for one of these trials (Milrod *et al*., 2007). For the other ‘very good’ trial (Shapiro *et al*., 1994), there were two possible conceptual replication attempts. Unfortunately, one attempt used a sub-clinical population, and the other did not corroborate the original result, particularly the large effect favouring CBT *vis-à-vis* dynamic therapy (Barkham *et al*., 1996; Barkham *et al*., 1999).

The non-replication of trials producing large effects is problematic for meta-analytic conclusions. Several such trials appeared in multiple meta-analyses from our database. Meta-analyses with more than two large effect trials reported the strongest evidence for treatment superiority. For example, Tolin (2010) found CBT superior to other treatments at post-treatment on measures of depression or anxiety (*d* = 0.22) and this analysis included four trials with large effects. Similarly, Cuijpers *et al*. (2016) reported evidence for the superiority of CBT (*d* = 0.29) on measures of depression at post-treatment and included four trials with large effects. By contrast, the meta-analyses in our database with only one or no large effect trials reported more modest findings, even for the same class of treatments (e.g. CBT) (Cuijpers *et al*., 2006, 2010*b*).

Before we discuss broader implications of these findings, we examine the most valid replication attempt from our results. The original trial known as the Second Sheffield Psychotherapy Project (SPP2), conducted by Shapiro *et al*. (1994), compared cognitive-behavioural psychotherapy to psychodynamic-interpersonal psychotherapy with two different durations (eight or 16 sessions) for the treatment of depression. In this study, the patients were treated in a research clinic at the University Sheffield.

In 1996, Barkham *et al*. (1996) sought to conceptually replicate the results of SPP2 in an applied setting as opposed to the special arrangements that existed in a speciality research clinic in a university setting, intentionally varying one aspect of the experimental arrangement. Thus, the intention and procedure align with the idea of a conceptual replication (Schmidt, 2009). The title of the Barkham trial clearly denoted their intention: ‘Outcomes of time-limited psychotherapy in applied settings: Replicating the Second Sheffield Psychotherapy Project’ (emphasis added). Moreover, Barkham *et al*. explicitly compared the results of the influential study and the replication, as summarised in online Supplementary Table S5. As the only trial that closely hewed to the idea of a conceptual replication in our investigation, it could serve as a model for researchers.

Recent reviews have expressed the need to increase the focus on replication in clinical social sciences (Tackett *et al*., 2019). We add to these efforts by highlighting how non-replicated findings influence superiority conclusions in psychotherapy. The trials with large effects in our review tended to have small samples, thus it cannot be ruled out that their effects were due to chance. At the same time, our unrestricted search suggests that replication attempts are rare regardless of trial effect or sample size. A recent investigation (Sakaluk *et al*., 2019) supports this conclusion, reporting ‘weak’ evidence for replicability across empirically supported psychotherapies. Unfortunately, a limitation of this study was its classification system, which grouped heterogeneous treatments and treatment comparisons.

Moving forward, replication should be a collective priority in psychotherapy research. A challenge is trial quality. High-quality trials appear to be the exception, not the rule in psychotherapy research (Cuijpers *et al*., 2010*a*). The case could be made that methodologically flawed trials should not be replicated. From this perspective, the dearth of replication for poor trials is expected, even desirable. However, if a low-quality trial is unworthy of a replication attempt, should it be admissible as scientific evidence in the first place? Clearly, low-quality trials should not serve as scientific evidence in a meta-analysis, and at the same time be exempt from replication standards. For example, Shaw (1977) and Shear *et al*. (2001) were two of the low-quality trials in our review, yet they appeared in multiple meta-analyses from our database (see Table 3). Unrepresentative therapists (one therapy, Shaw) and an unavailable treatment manual [Shear, personal communication, 7 March 2015] make these trials impossible to replicate.

Aside from trail quality, other challenges exist. Failure to replicate findings is often attributed to the lack of adherence to the treatment protocol (Laska *et al*., 2014). For example, when advocates of a particular treatment are confronted with a trial that fails to support the treatment, they often claim that the treatment was not given with adequate adherence to the manual (Laska *et al*., 2014; Wampold and Imel, 2015), an argument that attributes importance to the precise nature of treatments. According to this view – and one that we adopted for this investigation – the particular manual used to guide treatment delivery in a given trial must also be used in its replication. Some incremental steps towards addressing these challenges include (1) the development of authoritative replication criteria for clinical trials, and (2) stipulating quality benchmarks to aid research decisions to replicate trials.

The results of our investigation should be interpreted with caution. The replication criteria developed in Table 1 are the most logically consistent with the concepts of direct or conceptual replication in science writ large, but these criteria are not universally accepted for psychotherapy trials. In addition, our cut-off threshold (*d* = 0.80) for large effect trials is arbitrary, but by the same token, any cut-off value would be similarly arbitrary. Nevertheless, the conclusions that there are few replications of psychotherapy comparative studies seem robust (i.e. is not dependent on the cut-off chosen).

In conclusion, attempts to identify the most effective treatment for a particular disorder have not been successfully reproduced among trials showing the staunchest evidence. It seems wise to further investigate the replicability of psychotherapy trials. Especially if we are to have confidence in treatment guidelines premised on the assumption of treatment superiority.

## Footnotes

1 Effect size (*d* = 1.44) for Shapiro et al., 1994 was obtained directly from Tolin (2010) For 16 sessions, moderate depression, BDI at post-treatment. Statistics reported in Shapiro et al., (1994) does not allow verification of this effect.

## Supplementary material

For supplementary material accompanying this paper visit https://doi.org/10.1017/S2045796020000402. click here to view supplementary material

## Data

Clinical trial and meta-analytic databases for this review can be found at https://data.mendeley.com/drafts/.
