# Supplementary material for: Comparative clinical trials in psychotherapy: Have large effects been replicated?
Source: Epidemiol Psychiatr Sci. 2020 May 15;29:e128. doi: 10.1017/S2045796020000402 (PMC7232123; doi:10.1017/S2045796020000402)
Supplement: Supplementary file 1 [file S2045796020000402sup001.zip › Table_4-Replication_Attempts_Revised.docx]

Table 4. *Study* *characteristics of possible conceptual replication attempts*

| **Primary**  **trial** | **Replication**  **attempt** | **Tx** | **Dose** | **Form** | **Manual** | **Dx** | **Client sample** | ***N*** | **Therapist** | **Adhere Check** | **Outcome Measure** | **Results** |
| --- | --- | --- | --- | --- | --- | --- | --- | --- | --- | --- | --- | --- |
| *Latour & Cappeliez (1994)* | Watt & Cappeliez (2000) | Yes | Less | Yes | No | Yes | Yes | Same | No | Poorer | Same | Different |
| *Durham et al., (1994)* | Knekt et al. 2008^a,b^ | Yes | Same | Yes | No | No | Yes | Larger | Yes | Poorer | Same | Different |
|  | Leichsenring et al., (2009) | Yes | More | Yes | No | No | Yes | Same | Yes | Better | Same | Different |
|  | Arntz (2003) | Yes | No | Yes | Yes | Yes | Yes | Same | Yes | Better | Same | Different |
| *Gallagher-Thompson & Steffen (1994)* | Chaves et al., (2017) | Yes | No | No | No | Yes | No | Larger | No | Poorer | Same | Different |
|  | Gallagher-Thompson et al., (2003) | Yes | No | No | Yes | No | Yes | Larger | Yes | Same | Different | Same |
|  | Mohr et al., (2001) | Yes | Yes | No | Yes | Yes | No | Smaller | Yes | Same | Same | Marginally similar |
| *Klausner et al., (1998)* | Pretorious et al., (2008) | Yes | Yes | Yes | No | No | No | Smaller | No | Poorer | Different | Same |
| *Shaw (1977)* | Jacobson et al., (1996) | Yes | Yes | No | No | Yes | No | Larger | No | Better | Same | Different |
|  | de Jong, Treiber & Henrich (1986) | Yes | Same | No | No | No | No | Larger | No | Same | Same | Different |
|  | McNamara & Horan (1986) | Yes | Less | No | No | Yes | Yes | Larger | No | Better | Same | Similar |
|  | Wilson (1983) | Yes | Less | No | Yes | Yes | No | Larger | No | Same | Same | Different |
|  | Comas-Diaz (1981) | Yes | Less | No | No | Yes | No | Larger | No | Better | Same | Different |
| *Shapiro et al., (1994)* | Barkham et al., (1999) | Yes | Less | Yes | Yes | No | No | Same | Same | Same | Same | Different |
|  | Barkham et al., (1996) | Yes | Yes | Yes | Yes | Yes | No | Less | Yes | Poorer | Same | Marginally different |

*Tx = Did replication use the same treatment, Form = Format of treatment (i.e. individual versus group), Dx = diagnosis of client population, Therapist = Therapist with same level of training and experience as influential trial, Adhere Check = did the replication attempt utilize similar adherence checks, Outcome Measures = did replication evaluate outcomes on the same measure, Results = did conclusions of the replication corroborate influential trial.
