# Supplementary material for: Comparative clinical trials in psychotherapy: Have large effects been replicated?
Source: Epidemiol Psychiatr Sci. 2020 May 15;29:e128. doi: 10.1017/S2045796020000402 (PMC7232123; doi:10.1017/S2045796020000402)
Supplement: Supplementary file 1 [file S2045796020000402sup001.zip › Table_5-Exemplar_Replication_Comparison.docx]

Table 5. *Comparison between influential trial (SPP2; Shapiro et al., 1994) and conceptual replication attempt (CPP; Barkham et al., 1996)*

| **Question** | **SPP2** | **CPP** |
| --- | --- | --- |
| Is CB more effective than PI in the hands  of clinician-investigators holding no prior allegiance to CB? | Medium ES advantage (0.5) to CB but on BDI only | Large ES advantage (>. 8) to CB but on IIP only |
| Is CB more rapid in effects than PI? | Scant evidence of more rapid change in CB | No significant Treatment *x* Duration interaction effects |
| Do any differential effects of CB vs. PI vary according to the initial severity of depression? | No convincing evidence of differential response to CB or PI | No significant Severity *x* Treatment interactions effects |
| Is 16-session therapy more effective than 8-sessions therapy, and does any difference vary with initial severity of Depression | Weak evidence for superiority of 16 over 8 sessions but 16 more effective for high severity group | Superiority of 16 sessions over 8 sessions at EOT on BDI, SE, IIP; maintained at 3MFU and 1YFU on IIP only |

*Note.* Adapted from “Comparison of Findings Between CPP and SPP2,” by Barkham et al., 1996, *Journal of Consulting and Clinical Psychology, 64*(5), p. 1082. Copyright 1996 by the American Psychological Association. CPP = Collaborative Psychotherapy Project; SPP2 = Second Sheffield Psychotherapy Project; CB = Cognitive-Behavioral Treatment; PI = psychodynamic interpersonal treatment; ES = effect size; BDI = beck depression inventory; IIP = inventory of interpersonal problems; EOT = end of treatment; SE = self-esteem scale; 3MFU = 3 month follow-up; 1YFU = 1 year follow up.
